# Supplementary material for: Crawling and Gliding: A Computational Model for Shape-Driven Cell Migration
Source: PLoS Comput Biol. 2015 Oct 21;11(10):e1004280. doi: 10.1371/journal.pcbi.1004280 (PMC4619082; doi:10.1371/journal.pcbi.1004280)
Supplement: S1 Code — (ZIP) [file pcbi.1004280.s012.zip › release/tst/doc/html/misc_8h_source.html]

Tissue Simulation Toolkit: misc.h Source File


Tissue Simulation Toolkit
 0.1.4.1


- Main Page
- Namespaces
- Classes
- Files

- File List
- File Members

misc.h

Go to the documentation of this file.

1 /\*

2

3 Copyright 1996-2006 Roeland Merks

4

5 This file is part of Tissue Simulation Toolkit.

6

7 Tissue Simulation Toolkit is free software; you can redistribute

8 it and/or modify it under the terms of the GNU General Public

9 License as published by the Free Software Foundation; either

10 version 2 of the License, or (at your option) any later version.

11

12 Tissue Simulation Toolkit is distributed in the hope that it will

13 be useful, but WITHOUT ANY WARRANTY; without even the implied

14 warranty of MERCHANTABILITY or FITNESS FOR A PARTICULAR PURPOSE.

15 See the GNU General Public License for more details.

16

17 You should have received a copy of the GNU General Public License

18 along with Tissue Simulation Toolkit; if not, write to the Free

19 Software Foundation, Inc., 51 Franklin St, Fifth Floor, Boston, MA

20 02110-1301 USA

21

22 \*/

23 #define FALSE 0

24 #define TRUE 1

25 #define OK 1

26 #define REMARK 56

27

28

29 int ReadNumber(FILE \*file,int \*number);

30 int YesNoP(const char \*message);

31 char \*GetFileName(const char \*message,const char \*ftype);

32 int FileExists(FILE \*\*fp,const char \*fname,const char \*ftype);

33 int ReadDouble(FILE \*file,double \*number);

ReadDouble

int ReadDouble(FILE \*file, double \*number)

**Definition:** misc.cpp:88

GetFileName

char \* GetFileName(const char \*message, const char \*ftype)

**Definition:** misc.cpp:140

FileExists

int FileExists(FILE \*\*fp, const char \*fname, const char \*ftype)

**Definition:** misc.cpp:107

ReadNumber

int ReadNumber(FILE \*file, int \*number)

**Definition:** misc.cpp:72

YesNoP

int YesNoP(const char \*message)

**Definition:** misc.cpp:120


---

Generated on Thu Aug 14 2014 22:04:01 for Tissue Simulation Toolkit by  

 1.8.6
